# Direct measurement of interaction forces between bovine serum albumin and poly(ethylene oxide) in water and electrolyte solutions

**DOI:** 10.1371/journal.pone.0173910

**Published:** 2017-03-15

**Authors:** Sergio M. Acuña, José M. Bastías, Pedro G. Toledo

**Affiliations:** 1 Department of Food Engineering, University of Bío-Bío, Chillán, Chile; 2 Department of Chemical Engineering and Laboratory of Surface Analysis, University of Concepción, Correo 3, Concepción, Chile; Universite du Quebec a Trois-Rivieres, CANADA

## Abstract

The net interaction between a probe tip coated with bovine serum albumin (BSA) protein and a flat substrate coated with poly(ethylene oxide) (PEO) polymer was measured directly on approach in water and electrolyte solutions using AFM. The approach force curve between the two surfaces was monotonically repulsive in water and in electrolyte solutions. At pH ~5, slightly above the isoelectric point (pI) of BSA, and at large distances, the force was dominated by electrostatic repulsion between the oxygen atoms of the incoming protein with those belonging to the ether groups of PEO. Such repulsive force and range decreased in NaCl. Under physiological conditions, pH 6, BSA is definitely charged and the electrostatic repulsion with ether groups in PEO appears at larger separation distances. Interestingly, at pH 4, below the pI of BSA, the repulsion decreased because of an attractive, although weak, electrostatic force that appeared between the ether groups in PEO and the positively charged amino groups of BSA. However, for all solution conditions, once compression of PEO begun, the net repulsion was always dominated by short-range polymeric steric repulsion and repulsive enthalpy penalties for breaking PEO-water bonds. Results suggest that PEO in mushroom conformation may also be effective in reducing biofouling.

## Introduction

Microorganisms adhere to moist surfaces and develop biofilms that are responsible for several infectious diseases, some of which are device-related. Such biofilms pose a threat to water and food safety, and adversely affect the functioning of petroleum pipelines and aquatic flow systems, as well as the fabrication of textiles, contact lenses and medical implants. Thus, effective anti-biofilm technologies are in urgent need and should be a research priority, as established in recent reviews [1–4].

Chemical-based approaches, in which the surface chemistry of substrates is modified or the substrates are protected directly with an antibacterial coating, have proven very effective for repelling bacterial cells, preventing their attachment, or inactivating cells once they reach the surfaces. In particular, poly(ethylene oxide) (PEO), poly(oxyethylene) (POE), or poly(ethylene glycol) (PEG), -[CH_2_-CH_2_-O]_*n*_-, a water-soluble synthetic polymer, is widely used as an effective coating that affords surfaces resistant to biofilm formation [5–23]. PEO polymer chains attached to a surface, exist in two different conformations, the so-called mushroom structure at low chain grafting densities and the brush structure at higher chain grafting densities [11,12]. Both structures provide a steric barrier to the adhesion of proteins and microorganism, with the brush conformation being the most effective [12].

Considering that bacteria adhere to surfaces via adhesins that are comprised of proteins and/or polysaccharides, it has been of great interest to study the intermolecular interaction between proteins and the protective polymer coating [5,8,11,19,24,25]. However, if the PEO brush architecture is accomplished with flaws and nanoscale heterogeneities, these may induce bacterial fouling and cell adhesion long before protein adsorption occurs, see for instance [21]. Well-implemented PEO brushes should repel proteins, provided their height exceeds the range of electrostatic and van der Waals attraction, and their density is sufficiently high to avoid penetration by small proteins. The PEO mushroom architecture interacts with proteins through long-range electrostatic forces, which are repulsive when the liquid medium is above the isoelectric point (pI) of the protein and moderately repulsive below the pI [5,7,11,18–20]. Additionally, the interaction involves short-range repulsive forces, including polymeric steric, enthalpy penalties for breaking PEO-water bonds, and a hydrodynamic lubrication force due to the expulsion of solvent residing between the PEO coating and the protein [5,7,11,18–20].

The literature on the interaction between protein-covered surfaces and PEO layers is extensive, but the vast majority of studies have been done under physiological conditions. In this paper, we focus on directly measuring the force on approach of a probe tip coated with bovine serum albumin (BSA) and a flat substrate coated with PEO, in mushroom configuration, in the presence of NaCl at various concentrations and pH. The results are useful in themselves but specifically serve to verify the interaction forces and the components of the total net force regarding their sensitivity to salt and pH.

## Experimental

### BSA-coated glass slide and microsphere

A 20 μm diameter glass microsphere (Duke Scientific) and a glass microscope slide were rinsed with deionised water (EASYpure LF, 18.3 MΩ·cm), then washed with ethyl alcohol (analytical grade, JT Baker), and finally flushed with deionised water. The microsphere was glued to the free end of a tipless, V-shaped, 100 μm long, 0.6 μm thick Si_3_N_4_ cantilever (Veeco) with Norland optical adhesive 61 (Norland Products) according to the protocol described by Acuña and Toledo [26]. The spring constant of the cantilever was determined as 0.14 N/m by using a Dimension 3100 AFM (atomic force microscopy) microscope based on the standards provided by Park Scientific. Both the modified cantilever, after placing it in the micropositioner of the AFM microscope, and the microscope slide, were immersed in a solution containing 1 g/L BSA in 0.01 M NaCl at pH 5, 25°C for 4 h. Self-assembled monolayers (SAM) of BSA molecules formed spontaneously on the glass surfaces by adsorption. The topography and organisation of the SAM were determined by AFM.

### PEO-coated glass slide

A glass microscope slide (B&C) was coated with a stable polystyrene (PS) film by first grafting vinyl-terminated PS (M_w_ = 1100 Da, M_w_/M_n_ = 1.12, Polymer Source Inc.) to silicon atoms on the glass surface and then adsorbing PS (M_w_ = 740 Da, M_w_/M_n_ = 1.06, Polymer Source Inc.) from solution [27,28]. The PS-covered glass slide was then coated with poly(ethylene) oxide (PEO, M_w_ = 37800 Da, M_w_/M_n_ = 1.08, Polymer Source Inc.) using a Langmuir–Blodgett balance (KSV 3000, System 3). PEO was deposited by withdrawing the PS-covered slides from the water subphase at a constant surface pressure of 7 mN/m and a withdrawal speed of 3 mm/min. PEO deposition was repeated five times. The transfer ratio was 0.851.

### Topography

Glass slides coated with BSA and PEO were characterised with an AFM microscope (Dimension 3100, Digital Instruments/Veeco) equipped with a scanner capable of scanning areas of 100 × 100 μm^2^. Measurements were carried out in air at room temperature. Commercially available tapping mode tips were used on the cantilevers with a resonance frequency ranging from 350–400 kHz. The flat substrates were scanned at various resolutions, ranging from 50 × 50 μm^2^ to 2 × 2 μm^2^. The scans show overall similar patterns, however, the topographic features, root mean square (rms) roughness, height profile and average height, were measured in the highest resolution scan. The Digital Instruments Nanoscope IIIa v4.42 software was used for data acquisition and the WSxM 3.0 v8.3 software was used for image analysis [29].

### Contact angles

Water dynamic contact angle measurements were carried out using a KSV Sigma 700 tensiometer at room temperature. The BSA- and the PEO-coated glass slides were used as Wilhelmy plates dipped in pure water.

### Interaction force

The force between the BSA-coated microsphere of the modified cantilever and each of the PEO-coated glass slides was measured with a Multimode AFM-3 (Digital Instruments/Veeco) equipped with Nanoscope IIIa SPM control station (Digital Instruments/Veeco), fluid cell, silicone pad to isolate vibrations and acoustic cap (Digital Instruments/Veeco). The samples were handled with tweezers to avoid any contamination. Once the substrate and the modified cantilever were properly mounted, the fluid cell was flushed repeatedly with high-purity water and then with the electrolyte solution of interest. The system was set to reach equilibrium for few minutes before the probe and substrate approached each other. Data were obtained with the Nanoscope IIIa v4.42 instrument software (Digital Instruments/Veeco). Sample displacement was determined with a Z-piezoelectric crystal that was calibrated frequently with different step-height standards. These data were converted into force versus separation curves by using the well-established method of Ducker et al. [30,31] implemented in commercial software and in our own routine. Typically, 3584 data points were measured for each approach curve. The approach speed was 0.5 Hz. Reproducibility of the curves was ensured by performing at least three measurements at three different positions on the surface of each substrate. Once the reproducibility was verified, the definite force curves were obtained. Each curve reported here corresponds to the mean of at least 10 force curves measured at the same position. The approach force curves were measured in aqueous NaCl (Merck) solutions ranging from 10−4–10^−2^ M, pH 5.1, and also in pure water adjusted with 0.1 M HCl (Merck) to pH 4.0 and with 0.1 M NaOH (Merck) to pH 6.0. The standard deviation increased with electrolyte concentration, although it always remained small and negligible. The forces are reported normalised by the microsphere probe radius, namely, as an interaction energy between flat surfaces by virtue of Derjaguin’s approximation [32], as *F*(*D*) = 2*πRE* (*D*), where *F* is force, *D* is distance, *R* is probe radius, and *E* is energy per unit area.

## Results and discussion

**Fig 1:**
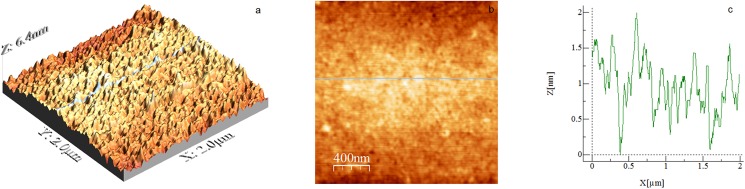
AFM image of a BSA-coated glass slide. Scan size is 2 × 2 μm^2^. (a) 3D image, (b) 2D image and c) height profile corresponding to the position signalled by the green line in (b).

Fig 1 shows a typical AFM image (2 × 2 μm^2^) of a BSA-coated glass slide after incubation with the BSA solution at 25°C, pH 5 for 4 h. The BSA concentration of 1 g/L was chosen slightly lower than the saturation point of protein adsorption on surfaces. The protein coating in Fig 1 is relatively uniform with no holes or aggregates dispersed over the surface. Table 1 shows that the BSA-adsorbed surface has an rms roughness of 3.6 Å and an average height of 16.2 Å. The roughness determined from AFM images of the microscope slide (not shown) was ∼1.4 Å rms (over 4 μm^2^ area). The image in Fig 1 is consistent with that for BSA adsorbed onto octadecyltrichlorosilane SAMs on silicon [33], but less comparable to BSA adsorbed on SAM-coated semiconductor wafers [34], which are rougher (respectively 24 and 27 Å rms). The contact angle measured here for water advancing across the BSA-coated glass slide is 69.1° (see Table 2), which is similar to the 73° reported independently by Follstaedt et al. [34] and Sánchez-González et al. [35].

**Table 1:** RMS roughness and mean height of a BSA-coated glass slide and a PEO-coated glass slide.

Substrate	Scan size (μm^2^)	RMS roughness (nm)	Average height (nm)
BSA coating	2 × 2	0.36 ± 0.03	1.62 ± 0.21
PEO coating	2 × 2	4.8 ± 0.18	10.42 ± 0.43

**Table 2:** Dynamic advancing and receding contact angles of water on a BSA-coated glass slide and a PEO-coated glass slide.

Substrate	*θ*_*A*_ (°)	*θ*_*R*_ (°)
BSA coating	69.1 ± 3.3	49.4 ± 2.2
PEO coating	100.1 ± 2.3	67.5 ± 1.8

**Fig 2:**
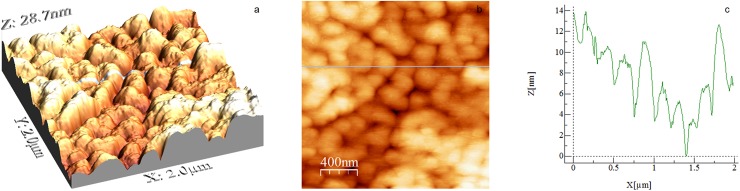
AFM image of a PEO-coated glass slide. Scan size is 2 × 2 μm^2^. (a) 3D image, (b) 2D image and c) height profile corresponding to the position signalled by the green line in (b).

Fig 2 shows a high-resolution AFM image (2 × 2 μm^2^) of a PEO-coated glass slide. The coating is uniform but highly porous with a roughness of 48 Å rms and an average height of 104 Å. According to Gombotz et al. [23], high molecular weight (> 1000 Da) PEO surfaces exhibit higher wettability, lower contact angles measured through the wetting liquid, and less protein adsorption than low molecular weight PEO surfaces. High molecular weight PEO surfaces, similar to the one used here, adopt the interacting mushroom conformation, according to Louguet et al. [20], which is confirmed by the AFM image in Fig 2. The average height of our PEO coating (104 Å) may represent the end-to-end distance of typical PEO chains. For a PEO surface of similar molecular weight, Louguet et al. [20] experimentally measured 175 Å and theoretically calculated 118 Å. The advancing and receding contact angles on PEO-coated glass slides were, respectively, 100.1 and 67.5° (see Table 2), revealing a surprisingly high hydrophobic surface. For a high molecular weight PEO, a hydrophilic surface is expected according to Gombotz et al. [23]. Our results differ from those of Roosjen et al. [10] and also from those of Gombotz et al. [23], who reported respective advancing and receding contact angles of 48 and 16° on PEO-coated glass and 41 and 16° on PEO-coated silica. The latter authors used PEO chains with a molecular weight several-fold smaller than used here, which explains the high degree of water wettability of their PEO-coated substrates. Our results are also at variance with those of Gombotz et al. [23], who found that the advancing contact angle on PEO surfaces, as a function of PEO molecular weight, decreased with PEO molecular weight, particularly between 200–1000 Da (from 51 to 37°). The latter authors used bis-amino PEO surfaces, which are more prone to wetting by water. Jo and Park [6] measured in one of their systems high advancing and receding contact angles (from 43 to 49.8° and from 18.4 to 24.4°, respectively) on PEO chains relatively similar to those used here. The high contact angles measured in our work are likely due to the hydrophobic PS coating of our glass slides before the final PEO coating.

**Fig 3:**
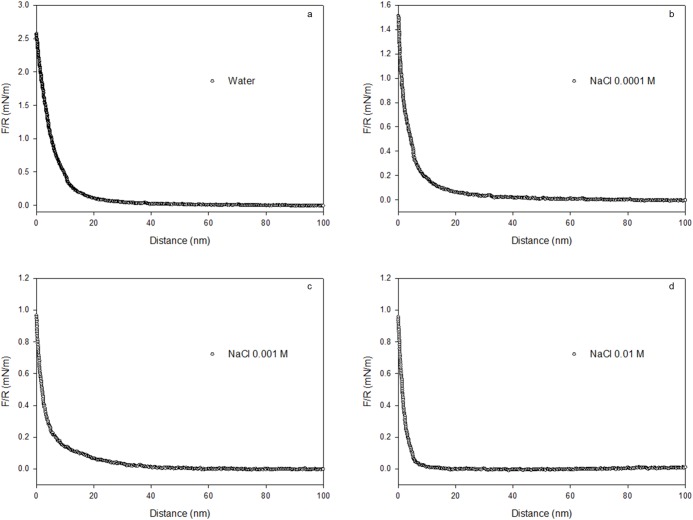
Approach force curves for the interaction between BSA and PEO in a) bidistilled water, and b) 0.0001 M, c) 0.001 M and d) 0.01 M NaCl. Each curve represents the average of ten independent AFM measurements taken from equivalent positions on the substrate.

The interaction force between the functionalised BSA-coated microsphere and the PEO-coated glass slide was measured in bidistilled water and in 10^−4^, 10^−3^ and 10^−2^ M NaCl solutions at pH ~5. The pH was slightly above the pI of the protein (pI 4.7 at 25°C, Ge et al. [33]). The reproducibility of the force curves obtained for each liquid at ambient conditions (Table 3), were verified by performing at least three measurements at three different points on the surfaces. The approach force curve (Fig 3), which is an average of ten curves measured at the same location, corresponding to each liquid at ambient conditions, reveals that the interactions measured are highly reproducible. The interaction is evidently dominated by a monotonic nonlinear repulsive net force, both in water and in the electrolyte solutions, for probe-substrate separation distances < 30 nm. Repulsion decreases exponentially as the separation distance between the interacting surfaces increases. Similar force-distance curves have been measured previously [11,19,36].

**Table 3:** Electrolyte type, concentration and pH of solutions used in the force measurements with AFM.

Electrolyte type	Concentration (M)	pH
None	~5 × 10^−6^^†^	6.0
		5.0
		4.0
NaCl	1 × 10^−4^	5.1
	1 × 10^−3^	5.1
	1 × 10^−2^	5.1

^†^ Bidistilled water (18.6 MΩ·cm) and a concentration roughly estimated as 10^−6^ M from material dissolved from the glassware.

**Fig 4:**
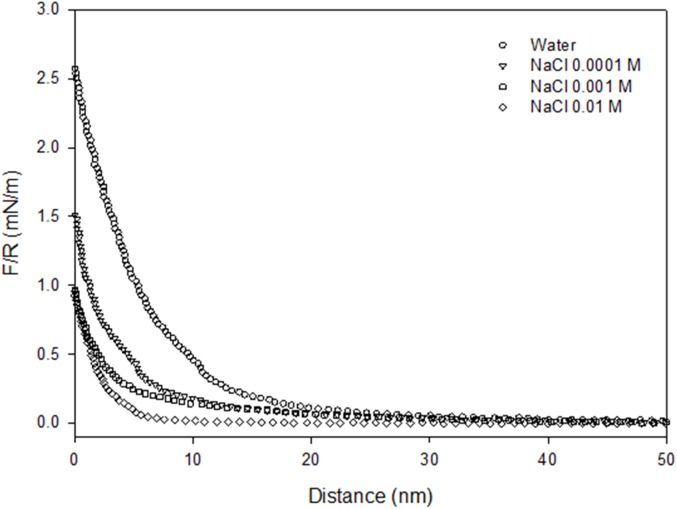
Effect of the electrolyte concentration on the interaction between BSA and PEO.

The conformation of BSA protein molecules and PEO polymer chains in the ambient fluid, in addition to the underlying physics of the net repulsion, should be considered to understand the repulsion between the BSA protein coating and the PEO polymer chains [21]. Fig 4 shows that the net repulsive force, and its range, decreases as the NaCl concentration increases in the ambient solution. At pH ~5 (~pI of BSA), the BSA molecules coating the probe become slightly ionised and, thus, the molecules expand a little due to repulsion between the terminal carboxylic groups, while the PEO chains that coat the glass slide, stretch away from the surface into the solution. Rixman et al. [11] propose two possible configurations of PEO in aqueous solutions. One is a “folded” trans-trans-gauche (ttg) configuration, in which the oxygen atoms in the ether groups form hydrogen bonds in various different ways. The second is an all-trans (ttt) planar zigzag configuration, in which the distance between the oxygen atoms in the ether groups is so large that a water molecule can only form one hydrogen bond with each oxygen atom in the PEO chains (see also Maron and Filisko [37]). Thus, the low water-wet condition of our PEO surface, aside from holes revealing underlying PS domains, may be a consequence of ttt arrangements. Local steric and electrostatic hindrances to water molecules or incoming proteins are not expected because the PEO chains do not have large side groups or fixed groups with explicit charge. This, in addition to the flexibility of the PEO chains, leads to a maximisation of the binding with water either in ttg or ttt configuration. The rupture of these PEO-water bonds, as the separation distance between the PEO and BSA surfaces becomes very small, contributes to the measured repulsive force in Figs 3 and 4. Another contribution to the repulsive force arises from polymeric steric interactions, particularly if the PEO chains are long enough to form brushes. Yet another contribution to the repulsive force in Figs 3 and 4, is the electrostatic repulsion between the oxygen atoms in the ether groups, and the negative charges on the PS at the bottom of the holes in the PEO-coated layer, and the negative charges on the incoming BSA. This repulsion increases with increasing compression of the polymeric layer. It is well-known that PS surfaces are not strictly neutral because they possess negative residual charges [38–40].

**Fig 5:**
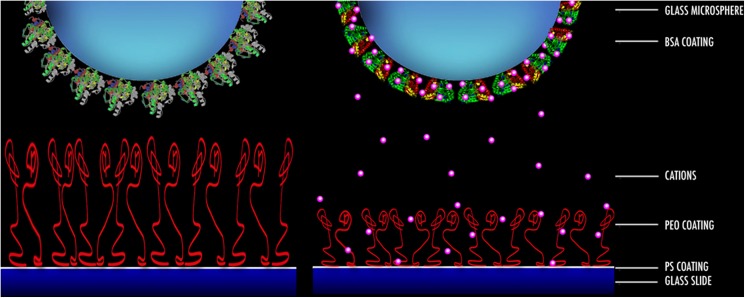
Schematic illustration of the interaction between BSA and PEO at pH above the pI of the protein in water (left frame) and in electrolyte solution (right frame). Shrinking of BSA and PEO has been intentionally exaggerated.

The presence of Na^+^ should not affect the conformation of the PEO chains because they have no explicit charge. However, the presence of two lone pairs of electrons on the oxygen atoms of their ether groups makes bonding with Na^+^ possible. Consequently, the polymer chains extend much less in the surrounding liquid medium in the presence of NaCl than water alone. Conversely, Na^+^ affects the BSA conformation by neutralising its charge. Thus, in the presence compared to the absence of Na^+^, the probe and sample approach each other over a longer distance without any net repulsion. In water (Figs 3 and 4), net repulsion occurs at distances as large as 30 nm apart, but in 0.1 M Na^+^, repulsion only occurs when the interacting surfaces are ~7 nm apart. In summary, the interaction between PEO and BSA surfaces at pH 5 is dominated by electrostatic repulsion at long separation distances, polymeric steric repulsion as the protein compresses the PEO polymer layer and repulsive enthalpy penalty for breaking PEO-water bonds at close PEO-BSA contact. The long-range electrostatic repulsion disappears in the presence of Na^+^ and the net repulsive force measured is dominated by the two short-range repulsive forces. Lastly, although the concentration of Na^+^ used in this study is very low, we should consider the effect of the Na^+^ ions (i.e. structure maker ions) on the structure of the water. Thus, at very short separation distances, another force opposing the approach may be operating, likely a hydrodynamic lubrication force enhanced by an increased viscosity due to the maker ion. Fig 5 shows a schematic of the BSA-PEO interaction at a pH above the pI of the protein.

**Fig 6:**
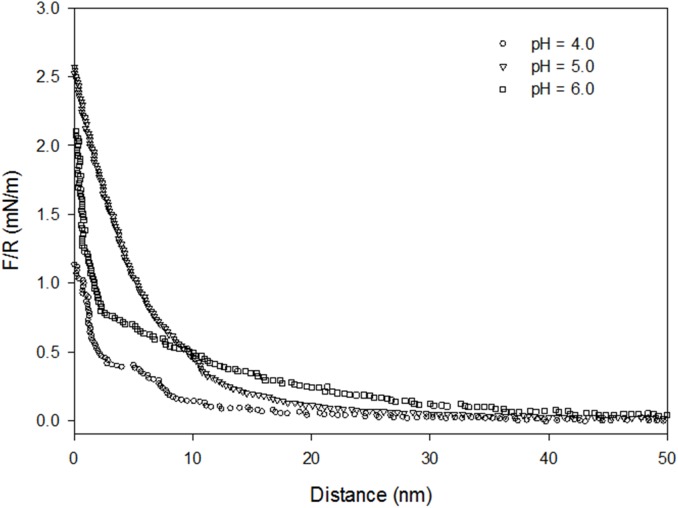
Effect of pH on the interaction between BSA and PEO.

The interaction force between the functionalised BSA-coated microsphere and the PEO-coated glass slide was also measured at pH 4 and 6 in bidistilled water (Fig 6). Under physiological conditions, pH 6, the BSA is definitely charged and the long-range electrostatic repulsion with the oxygen atoms of the ether groups in the PEO chains appears at separation distances of 50 nm and above. Such electrostatic repulsion dominates the interaction up to separation distances of 10 nm, at which, the polymeric steric repulsion and repulsive enthalpy penalty for breaking PEO-water bonds dominate. At pH 6, the inner repulsion between the carboxylic groups of BSA leads to slight expansion of the protein molecules, yet the normal globular form of BSA is maintained [41,42], which enhances the net repulsion with the PEO coating. Interestingly at pH 4 i.e. below the pI of the BSA, at high separations, the net repulsion decreases, the BSA-PEO electrostatic repulsion does no longer seem to be operating, instead an attractive although weak electrostatic force appears between the positive charges in the amino groups of the protein and the oxygen atoms of the ether groups in the PEO chains. However, the inner repulsion between the amino groups of the BSA leads to unfolding/expansion of the protein molecules [41,42]. Regardless of whether the unfolding is partial or full, it mitigates any attraction between the protein and the PEO chain. At very short separation distances (below 10 nm), the BSA and PEO surfaces oppose the compression by displaying strong polymeric steric repulsion and incurring high enthalpy penalties for disturbing hydration layers. However, unexpectedly, the strongest net repulsion between BSA and PEO surfaces at the pI occurs near contact. In this latter condition, the BSA protein is globular and very compact and, we speculate, very capable of developing thicker and more highly ordered hydration layers. Thus, as the surfaces of BSA and PEO come into close contact, the desolvation penalty dominates.

## Conclusions

The net nanoscale interaction between a probe tip coated with BSA protein and a flat substrate coated with a high molecular weight PEO polymer was measured directly on approach in water and electrolyte solutions using AFM. The protein coating was fairly uniform with neither holes nor any aggregates dispersed over the surface. Also, as expected, the wettability by water was low. Topographic maps revealed that the polymer coating was also uniform but highly porous, with high roughness and an average height of the order of the end-to-end distance of typical, high molecular weight PEO chains. As envisaged, the topographic maps also showed that the PEO chains adopted the interacting mushroom conformation. Unexpectedly, however, the coating was not water-wet, suggesting that the PEO polymer adopted a flat configuration that did not maximise hydrogen bonding with water. The approach force curve between the protein and PEO surfaces showed that the measurement was highly reproducible and that the interaction was dominated by a monotonic, nonlinear, repulsive net force, both in water and in electrolyte solutions. At pH ~5, slightly above the pI of the protein, the net repulsion force appeared at 30 nm and increased as the probe-sample separation distance decreased. The force was dominated by electrostatic repulsion between the oxygen atoms in the ether groups, and the negative charges on the PS at the bottom of the holes in the PEO coating layer, and the weak negative charges on the incoming BSA protein. At small separation distances (< 10 nm), compression of the polymeric layer begun. Thus, the net repulsion was dominated by polymeric steric repulsion and repulsive enthalpy penalties for breaking PEO-water bonds. The repulsive net force and its range decreased as the NaCl concentration in the ambient solution increased. The presence of Na^+^ did not significantly affect the conformation of the PEO chains because they had no explicit charge but did affect the conformation of the BSA protein. As the Na^+^ concentration increased, it neutralised the charge of the BSA protein, allowing both surfaces to approach from a longer distance without any net repulsion. At pH 6, the surface of BSA is electrically more negative and the long-range electrostatic repulsion with the oxygen atoms of the ether groups in the PEO chains appeared at separation distances of 50 nm and above Such electrostatic repulsion dominated the interaction up to separation distances of 10 nm, at which, the polymeric steric repulsion and repulsive enthalpy penalty for breaking PEO-water bonds controlled the interaction. Interestingly, at pH 4 i.e. below the pI of BSA, the net repulsion decreased and the electrostatic repulsion no longer appeared to be operating. Instead, an attractive, although weak electrostatic force, appeared between the positive charges in the amino groups of the protein and the oxygen atoms of the ether groups in the PEO chains. The surfaces opposed the compression at short separation distances by displaying polymeric steric repulsion and incurring enthalpy penalties for disturbing hydration layers. Unexpectedly, the strongest net repulsion between BSA and PEO near contact was at the pI. We speculate that the BSA protein, being highly compact at the pI, was able to develop thicker or more ordered hydration layers or both, and thus, as the surfaces of BSA and PEO come into close contact the desolvation penalty dominated. The results suggest that PEO may be effective in reducing microbial adhesion.
